# Radiation Necrosis with Proton Therapy in a Patient with Aarskog-Scott Syndrome and Medulloblastoma

**DOI:** 10.14338/IJPT-21-00013.1

**Published:** 2021-07-29

**Authors:** Vidya Puthenpura, Nicholas J. DeNunzio, Xue Zeng, Drosoula Giantsoudi, Mariam Aboian, David Ebb, Kristopher T. Kahle, Torunn I. Yock, Asher M. Marks

**Affiliations:** 1 Section of Pediatric Hematology and Oncology, Department of Pediatrics, Yale University School of Medicine, New Haven, CT, USA; 2 Department of Radiation Oncology, Massachusetts General Hospital/Harvard Medical School, Boston, MA, USA; 3 Department of Genetics, Yale University School of Medicine, New Haven, CT, USA; 4 Department of Neurosurgery, Yale University School of Medicine, New Haven, CT, USA; 5 Section of Neuroradiology and Nuclear Medicine, Department of Radiology, Yale University School of Medicine, New Haven, CT, USA; 6 Department of Pediatric Hematology/Oncology, Massachusetts General Hospital/Harvard Medical School, Boston, MA, USA

**Keywords:** brainstem radiation necrosis, medulloblastoma, Aarskog-Scott syndrome, hyperbaric oxygen therapy

## Abstract

**Purpose:**

Medulloblastoma is known to be associated with multiple cancer-predisposition syndromes. In this article, we explore a possible association among a patient's Aarskog-Scott syndrome, development of medulloblastoma, and subsequent brainstem radiation necrosis.

**Case Presentation:**

A 5-year-old male with Aarskog-Scott syndrome initially presented to his pediatrician with morning emesis, gait instability, and truncal weakness. He was ultimately found to have a posterior fossa tumor with pathology consistent with group 3 medulloblastoma. After receiving a gross total resection and standard proton beam radiation therapy with concurrent vincristine, he was noted to develop brainstem radiation necrosis, for which he underwent therapy with high-dose dexamethasone, bevacizumab, and hyperbaric oxygen therapy with radiographic improvement and clinical stabilization.

**Conclusion:**

Based on several possible pathologic correlates in the FDG1 pathway, there exists a potential association between this patient's Aarskog-Scott syndrome and medulloblastoma, which needs to be investigated further. In patients with underlying, rare genetic syndromes, further caution should be taken when evaluating chemotherapy and radiation dosimetry planning.

## Introduction

Medulloblastoma is the most common pediatric central nervous system malignancy [1]. The mainstay of treatment is surgical resection, followed by radiation therapy and adjuvant chemotherapy [2, 3]. The emergence of proton beam radiation has been critical for medulloblastoma therapy because of its ability to spare damage to healthy tissue, resulting in a reduction in both short-term and long-term side effects [3, 4]. Brainstem radiation necrosis, however, has occurred in the settings of both photon and proton radiotherapy [5].

Medulloblastoma is associated with several cancer predisposition syndromes, including Li-Fraumeni syndrome, nevoid basal cell carcinoma syndrome, and familial adenomatous polyposis [6]. Newer sequencing technologies allow identification of underlying cancer predispositions or other syndromes that would have been missed historically [7, 8]. In this case report, we present a patient with Aarskog-Scott syndrome who developed medulloblastoma with subsequent brainstem radiation necrosis.

## Case Presentation

A 5-year-old male with Aarskog-Scott syndrome, gastroesophageal reflux, and beta thalassemia minor initially presented with a 2-month history of morning emesis and decreased oral intake. He had subsequent difficulties with balance, truncal and extremity weakness, and a wide-based gait. His medical history, as part of his Aarskog-Scott syndrome, was significant for small stature and decreased weight, tracking under the first percentile for his age. His family history was significant for a father with history of childhood seizures and a younger brother who also has Aarskog-Scott syndrome.

Initial exam was significant for 4/5 strength in all extremities, truncal ataxia with a wide-based gait, and dysmetria. Magnetic resonance imaging (MRI) showed a 4.1 × 5.7 × 6.3-cm, heterogeneously enhancing tumor in the fourth ventricle, with obstructive hydrocephalus. An MRI of the spine and a lumbar puncture showed no signs of distant disease. The patient underwent gross total resection of the tumor. Pathology showed medulloblastoma, classic variant, World Health Organization grade IV, MYC/MYCN non-amplified, with loss of heterozygosity secondary to copy number gains of chromosome band 1q, 7, 9, and 14, as well as a copy number loss of chromosome band 16q, overlapping with the group 3 subtype [9].

**Figure 1.**
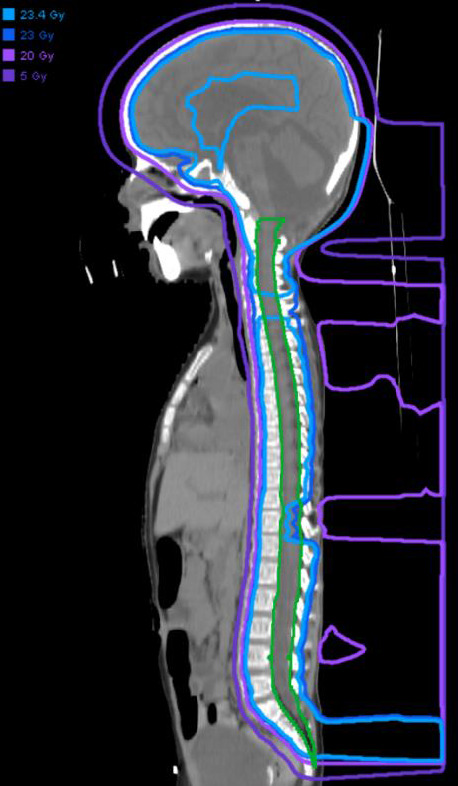
Craniospinal irradiation phase dosimetry. The radiation target is the whole brain and spinal canal (dark green line) inclusive of the thecal sac. The doses received by these regions are shown by a series of isodose lines for the 5 Gy (dark purple), 20 Gy (light purple), 23 Gy (dark blue), and 23.4 Gy (light blue) dose levels. Note the lack of low-dose radiation received by structures anterior to the spinal canal when using a proton beam that enters from the posterior surface.

**Figure 2.**
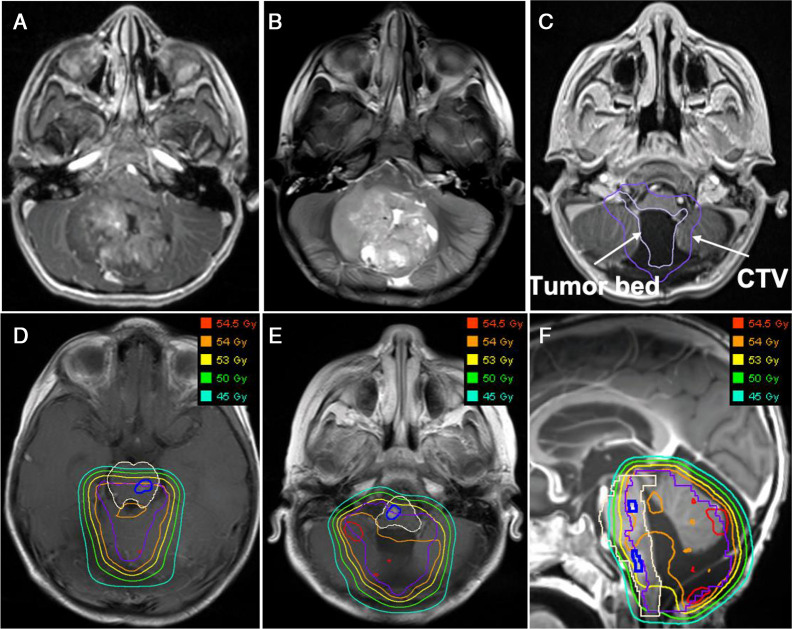
Boost phase dosimetry. (A–C) Representative magnetic resonance imaging (MRI) contrast-enhanced T1 (A) and T2 (B) images of the tumor in the preoperative setting and tumor bed in the postoperative setting (C, light purple). Note the extensive contact between the tumor and brainstem in the axial plane accompanied by brainstem displacement and compression. A margin was added to the known at-risk tissue volume to account for microscopic disease spread, also known as a clinical target volume (CTV; dark purple). (D–F) The doses received by the brainstem (cream) and neighboring tissues at the level of the superior and inferior lesions (dark blue) are shown by a series of isodose lines in transverse (D and E) and sagittal (F) views for the 45 Gy (aqua), 50 Gy (green), 53 Gy (yellow), 54 Gy (orange), and 54.5 Gy (red) dose levels.

Four days after the surgery, the patient was diagnosed with severe posterior fossa syndrome with poor global muscle tone, ataxia, mutism, and dysphagia. Subclinical seizures were confirmed by video electroencephalogram. A literature search revealed no contraindication or increased sensitivity to radiotherapy associated with Aarskog-Scott syndrome. His radiotherapy consisted of 23.4 GyRBE to the craniospinal axis (**Figure 1**) with an additional 30.6 GyRBE to the tumor bed, for a total dose of 54 GyRBE (**Figure 2**) with weekly vincristine at 1.5 mg/m^2^. Maximum and D_50_ (median) doses to the patient's brainstem were 54.4 GyRBE and 53.7 GyRBE, respectively, well within published guidelines for healthy brainstem tolerance [10–13]. Modest improvements were made in strength, mobility, and mutism with intensive rehabilitation.

**Figure 3.**
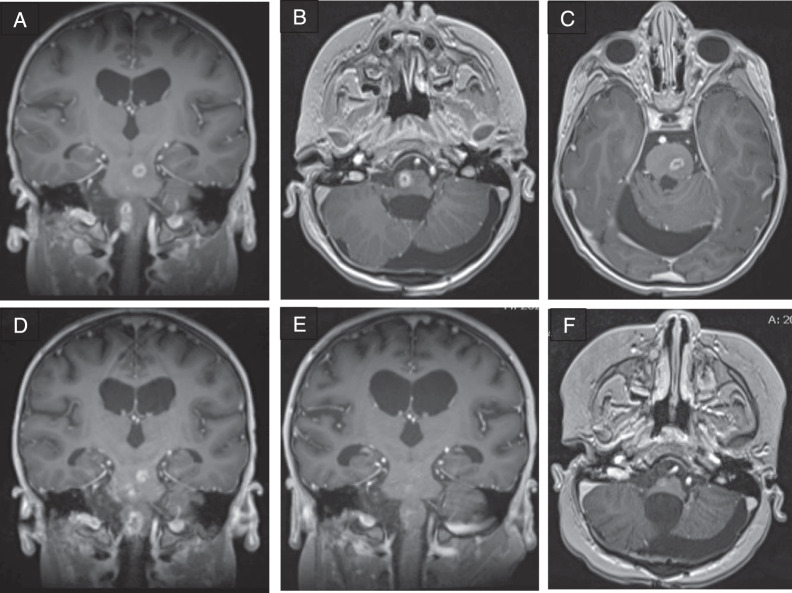
Development of rim enhancing lesions within the brainstem 5 months after radiation therapy. (A–C) Postgadolinium T1-weighted imaging approximately 5 months after radiation. (D) Postgadolinium T1-weighted imaging 6 weeks after initial imaging. (E and F) Postgadolinium T1-weighted imaging after completion of bevacizumab therapy.

He received his first 6-week cycle of maintenance chemotherapy with cisplatin, lomustine, and vincristine per ACNS0331 [14], cycle A prior to being discharged to home. Adjuvant chemotherapy continued with another A cycle, and then a 4-week B cycle comprising cyclophosphamide with Mesna and vincristine. Routine MRI, at 21 weeks after radiotherapy completion, revealed new, rim-enhancing lesions with central necrosis and surrounding edema in the left upper, middle, and lower pons, as well as in the right medulla and cervicomedullary junction, measuring up to 1 cm (**Figure 3A**–**3C**). All lesions were within the tumor-bed boost portion of the radiotherapy field and were judged to be consistent with radiation injury, although the patient had not manifested any neurologic deterioration. Further chemotherapy was held, and the patient was started on dexamethasone.

An MRI performed 6 weeks later showed lesion progression (**Figure 3D**). The patient developed worsening hemiparesis in the following days. Dexamethasone was continued, and bevacizumab was initiated at 10 mg/kg every 2 weeks for 6 doses [15, 16]. Mild improvement in symptoms occurred, and radiographic stability was observed on the following MRI 2 months later. Radiographic improvement continued after completion of bevacizumab therapy, with enhancing lesions subsequently subsiding (**Figure 3E** and **3F**); however, his neurologic deficits persisted.

Daily hyperbaric oxygen therapy was initiated 6 weeks later [17–20]. During this treatment, mild clinical improvement was reported. To date, the patient continues to have slow clinical improvement.

## Methods and Results

Genomic DNA of the patient, his father, mother, and younger brother was isolated from blood, and whole-exome sequencing was performed, as previously described [21]. Exon capture was performed using the IDT xGen Exome capture kit (Integrated DNA Technologies, Coralville, Iowa), followed by 101 base paired–end sequencing on the HiSeq 4000 platform (Illumina, San Diego, California). Sequence reads were aligned to the human reference genome GRCh37/hg19 using the BWA-MEM software and further processed to call variants, following the GATK best practices workflow [22, 23]. Variants annotated with ANNOVAR and MetaSVM software were used to predict the deleteriousness of nonsynonymous variants (herein, referred to as D-mis) [24, 25]. All variants covered by independent-aligned sequencing reads with a depth of 8× or greater were visualized in silico to eliminate false-positives.

To identify potential causal mutations for the disease phenotypes in the affected siblings, we filtered for rare, damaging mutations shared by both individuals. A non–frameshift deletion (c.2729_2746del, p.910_916del) in *FGD1* was identified in both siblings, which was transmitted from their unaffected mother. *FGD1* encodes “FYVE, RhoGEF and PH domain containing 1.” X-linked recessive variants in *FGD1* have been associated with Aarskog-Scott syndrome (OMIM: 305400) [26], which is consistent with the phenotype and the inheritance model observed in this family. Variants in this domain have previously been associated with Aarskog-Scott syndrome [27–29].

## Discussion

Despite radiotherapy dosimetry being well within published brainstem constraints for healthy tissue, this patient suffered radiation-related injury causing neurologic decline [10–13]. Dose heterogeneity in a radiation plan is unavoidable, but in this case, there were very few and mild hot spots.

Importantly, the pediatric radiation oncology community is increasingly recognizing that brainstem tolerance may be less with proton radiotherapy than it is with photon radiotherapy. The current Children's Oncology Group ependymoma study (ACNS0831) [30] uses lower brainstem dose constraints for proton radiotherapy compared with photon radiotherapy, a topic recently addressed at a workshop sponsored by the National Cancer Institute [10]. Nonetheless, this patient was treated with doses meeting those newer and lower brainstem dose constraints and still suffered symptomatic brainstem injury.

**Figure 4.**
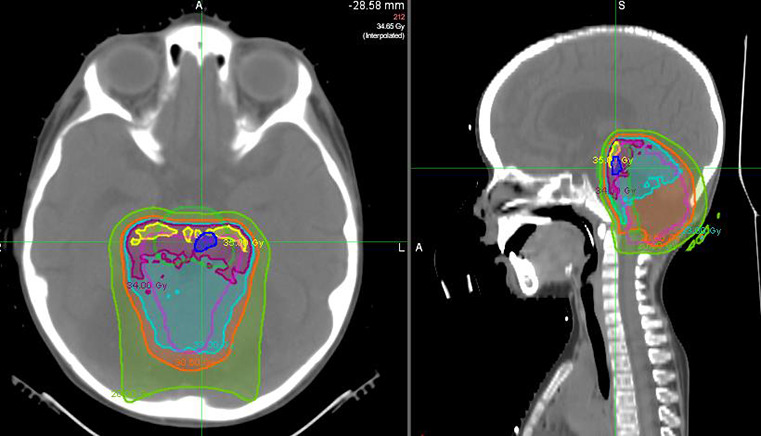
Relative biologic effectiveness (RBE) modeling overlay using a/b = 3. The brainstem (lime green) and superior contrast-enhancing brainstem lesion (royal blue) are outlined. The RBE contours are shown for 20 Gy (lime green), 30.6 (orange), 33 (aqua), 34 (magenta), and 35 (yellow). The area of brainstem injury partially overlaps the modeled area of highest RBE that, when added to the craniospinal irradiation (CSI) dose, equates to 57.4–58.4 Gy.

Integral to this discussion is consideration of the linear energy transfer (LET) and relative biologic effectiveness (RBE)–weighted dose distributions. The LET distribution here is typical for patients treated with this technique. The RBE depends heavily on the a/b ratio, which was 3 in this case (**Figure 4**). This RBE model attempts to account for the biological effective dose. Although it appears that the injury occurred in regions of higher RBE, it does not occur in the region of highest LET, and previous reports have been inconclusive. However, the LET differences across the proton-dose distribution may partially account for these unexpected findings. Better models for LET or RBE are needed to truly understand the potential effect on the patient. Newer radiation-planning systems are testing the incorporation of LET into the model, which could help diminish the apparent increased biological effective dose seen in certain parts of the dose distribution.

Many patients are treated with similar RBE/LET dose distributions and do not develop injuries. Thus, factors contributing to host radiosensitivity are also likely contributory. It behooves us to consider those factors that may have predisposed this patient to radiation sensitivity or injury and to consider those factors moving forward as we treat patients with genetic syndromes, blood dyscrasias, nutritional deficiencies, or other comorbidities that may impair growth and healing [31].

In this case, it is unclear what genetic components of Aarskog-Scott may have had a role in sensitivity. The *FGD1* gene implicated in Aarskog-Scott syndrome codes for a protein that activates Cdc42. This GTPase is important in cell signaling and is involved with many functions, including remodeling of the extracellular matrix and regulation of cell growth [32, 33]. The upregulation of Cdc42 is also associated with increased invasion of medulloblastoma, potentially making these tumors more aggressive [34, 35]. Review of the literature did not reveal any cases of patients with Aarskog-Scott who received proton or photon radiation.

This patient's relatively poor radiotherapy tolerance could also be related to phenotypic manifestations of his underlying syndrome, namely, his nutritional status and growth delay. He was < 1% of expected weight and height for his age, and patients with an impaired nutritional status may be predisposed to injury and have more difficulty repairing any sustained injury [36].

## Conclusion

It is likely that multiple factors, including an underlying syndrome and poor nutritional and growth status, contributed to this patient's high toxicity to treatment. When treating a child with a genetic syndrome, failure to thrive, and/or nutritional deficiencies, additional care must be taken in developing the radiotherapy plan. If radiation-related injury develops during adjuvant chemotherapeutic treatment, we recommend that treatment be halted immediately with early initiation of anti-inflammatory agents to avoid neurologic decline, regardless of symptoms at the time. Chemotherapy administration can cause further deterioration. Only when imaging findings of brainstem radiation necrosis resolve and there is no further clinical deterioration should chemotherapy be reinstated, if at all. Prolonged steroid and bevacizumab use is typically needed when clinically significant injury is present. Interestingly, hyperbaric oxygen therapy appears to have had some benefit for this patient and merits consideration going forward.
